# Supplementary material for: Early Arterial Intimal Thickening and Plaque Is Related with Treatment Regime and Cardiovascular Disease Risk Factors in Young Adults Following Childhood Hematopoietic Stem Cell Transplantation
Source: J Clin Med. 2020 Jul 13;9(7):2208. doi: 10.3390/jcm9072208 (PMC7408962; doi:10.3390/jcm9072208)
Supplement: Supplementary file 1 [file jcm-09-02208-s001.pdf]

Supplemental Material Sundholm et al. 2020, Early vascular ageing after childhood HSCT

Supplemental Table 1. Clinical characteristics of hematopoietic stem cell transplantation (HSCT) survivors.

n =62	Mean; Median; N	SD; Q1-Q3; %
Age at diagnosis [years]	8.1	3.0-12.0
Follow-up time [years]	17.5	14.1-23.0
Age at follow-up [years]	25.9	21.1- 30.1
Diagnosis		
Leukemia [n]	31	50 %
Acute Lymphocytic leukemia	26	42 %
Acute Myeloid leukemia	5	8 %
High-risk Neuroblastoma	18	29%
Severe Aplastic Anemia [n]	6	10 %
Other [n]	7	11 %
Treatment regime		
TBI [n]	46	74%
TBI dose [Gy]	10	10-12
Additional Gonadal Irradiation [n]	5	8%
Gonadal Irradiation Dose [Gy]	24	14-24
Additional CNS Irradiaton [n]	7	11%
CNS Irradiation dose [Gy]	16	6-26
Cyclophosphamides [n]	42	68%
CED [mg/m2]	5541	2000-23183
Anthracyclines [n]	41	66%
Cum.dose Anthracyclines [mg/m^2]	165	40-500
**HSCT-donor type**		
Autologous HSCT	19	31%
Allogenic HSCT	43	69%
HLA-identical sibling or other relative [n]	25	40%
HLA-identical, unrelated [n]	11	18%
Alternative donor [n]	6	10%
Graft vs Host Disease (GVHD)		
Acute GVHD [n]	33	53%
Acute GVHD (grade 3-4) [n]	10	16%

Chronic GVHD ever [n]	19	31%
Chronic GVHD ever (grade 2-3) [n]	6	10%
Chronic GVHD at follow-up [n]	10	16%
Chronic GVHD at follow up (grade 2-3) [n]	5	8%

Supplemental Table 1. Background data presenting HSCT survivor's primary diagnoses and treatments. CED – Cyclophopshamide equivalent doses; CNS – Central nervous system; Cum.dose – Cumulative dose; GVHD – Graft versus host disease; HLA – Human leukocyte antigen; HSCT – Hematopoietic stem cell transplantation; TBI – Total body irradiation.

Supplemental Table 2. ANCOVA-model assessing age-related increase of carotid intima-media thickness in HSCT survivors and controls.

**Dependent variable**	**n**	**R^2**	**Model p-value**
Carotid intima-media thickness [µm]	104	0.597	<0.001
**Independent variables**	**β**	**CI95%**	**p-value**
Constant	334.9	241.7;428.0	
Age [years]	3.4	0.0;6.8	0.053
Disease [0 = Control, 1 = HSCT]	-71.0	-196.5;54.4	0.264
*Age * Disease*	5.5	0.9;10.1	0.020

Supplemental Table 2. ANCOVA-model assessing difference in age-related increase of carotid intima-media thickness in HSCT-survivors and controls. HSCT- Hematopoietic stem cell transplantation.

Supplemental Table 3. Comparison of plaque and intimal thickening stratified for radiation therapy exposure.

	HSCT Non-TBI	HSCT TBI	HSCT TBI+Boost	Control	p-value
**Plaques**					
Any Plaques [y/n]	2/16	16/46***	-	2/44	0.001
Carotid Plaques [y/n]	1/16	13/39**	1/7	2/44	0.002
Femoral Plaques [y/n]	1/16	4/41	3/5**	0/44	0.001
**Intimal thickening**					
Any intimal thickening [y/n]	4/15*	14/43***	-	1/43	<0.001
Femoral [y/n]	1/15	6/38*	1/5	0/44	0.018

Supplemental Table 3. Prevalence of plaques and intimal thickening in HSCT stratified for radiation therapy exposure with subanalyses for patients exposed to local boost radiation therapy of gonads in femoral artery comparisons and the central nervous system for carotid artery comparisons. P-values represent results for Fisher-Freeman-Halton Exact-test, asterisks represent post hoc pair wise Fisher's exact tests with Bonferroni adjusted significance levels. * – differs significantly at $p < 0.05$-level compared to controls; ** – differs significantly at $p < 0.01$-level compared to controls; ***p – differs significantly at $p < 0.001$-level compared to controls; TBI – total body irradiation.

Supplemental Table 4. Comparison of vascular parameters between TBI-exposed and unexposed HSCT survivors.

	**Non-TBI**		**TBI**			Adjusted for Age & BSA	
	n=16		n=46			TBI – non-TBI	
	Mean	SD	Mean	SD	p-value	ΔMean	CI95%
Common carotid artery							
LD [mm]	5.59	0.73	5.32	0.47	0.093	-0.15	-0.40;0.11
IMT [mm]	0.47	0.14	0.50	0.10	0.339	0.03	-0.03;0.09
Radial artery							
LD [mm]	1.95	0.36	1.81	0.37	0.207	-0.07	-0.27;0.13
IMT [mm]	0.16	0.03	0.16	0.03	0.783	0.01	-0.01;0.02
AT [mm]	0.08	0.02	0.07	0.02	0.602	0.00	-0.01;0.01
Brachial artery							
LD [mm]	3.51	0.74	3.46	0.75	0.822	0.14	-0.17;0.44
IMT [mm]	0.17	0.04	0.19	0.05	0.275	0.02	0.00;0.04
AT [mm]	0.12	0.03	0.13	0.02	0.714	0.00	-0.01;0.01
Femoral artery							
LD [mm]	6.79	0.98	6.67	1.12	0.725	-0.02	-0.57;0.53
IMT [mm]	0.36	0.09	0.37	0.09	0.679	0.01	-0.03;0.06
AT [mm]	0.24	0.09	0.27	0.08	0.181	0.02	-0.03;0.07
Intimal thickening	N	%	N	%	p-value		
Any arteries [n]	4	27%	14	32%	0.756		
Femoral artery [n]	1	7%	7	16%	0.666		
Radial artery [n]	2	13%	7	16%	1.000		
Plaques	N	%	N	%	p-value		
Any plaques [n]	2	13%	16	36%	0.114		
Carotid Plaque [n]	1	7%	14	32%	0.088		
Femoral Plaque [n]	1	7%	7	16%	0.668		
Arterial stiffness	Mean	SD	Mean	SD	p-value		
CBSI	4.8	1.3	5.8	1.7	0.056	1.21[b]	0.33;2.10
CDC [%/10mmHg]	5.0	1.5	4.2	1.4	0.096	-0.97[a]	-1.72;-0.21
Carotid-femoral PWV [m/s]	8.7	1.5	8.8	1.4	0.904	-0.11	.-1.45;1.24
Carotid-radial PWV [m/s]	8.4	1.0	9.7	2.0	0.109	1.18	-0.56;2.91

Supplemental Table 4. Comparison of vascular parameters between TBI-exposed and unexposed HSCT survivors. PWV-data was available for HSCT cohort 1 only. AT – Adventitia thickness; BSA – Body-surface area; CBSI – Carotid β-stiffness index; CDC – Carotid distensibility coefficient; CI95% – 95% confidence interval; IMT – Intima-media thickness; LD – Lumen diameter; PWV – Pulse-wave velocity. [a] – significant at 0.05-level; [b] – significant at p-0.005 level.

Supplemental Table 5. Comparison of vascular parameters between autologous HSCT and allogenic HSCT survivors.

	**Autologous HSCT** *n=19*		**Allogenic HSCT** n=43			Adjusted for Age & BSA Autologous – Allogenic	
	Mean	SD	Mean	SD	p-value	ΔMean	CI95%
Common carotid artery							
LD [mm]	5.01	0.32	5.52	0.59	0.004	-0.10	-0.36;0.16
IMT [mm]	0.47	0.01	0.51	0.12	0.280	0.01	-0.05;0.07
Radial artery							
LD [mm]	1.63	0.29	1.96	0.36	0.001	-0.18	-0.37;0.02
IMT [mm]	0.15	0.03	0.170	0.03	0.030	0.00	-0.02;0.01
AT [mm]	0.08	0.01	0.07	0.02	0.026	0.01[a]	0.00;0.02
Brachial artery							
LD [mm]	2.80	0.49	3.78	0.63	<0.001	-0.57[c]	-0.84;-0.29
IMT [mm]	0.16	0.03	0.20	0.05	0.001	0.00	-0.02;0.02
AT [mm]	0.12	0.02	0.13	0.03	0.230	0.00	-0.02;0.01
Femoral artery							
LD [mm]	5.82	0.72	7.06	0.99	<0.001	-0.78[c]	-1.32;-0.24
IMT [mm]	0.31	0.04	0.39	0.09	<0.001	-0.05[a]	-0.09;-0.06
AT [mm]	0.25	0.09	0.27	0.08	0.570	-0.02	-0.07;0.03
Intimal thickening	N	%	N	%	p-value		
Any arteries [n]	5	26%	13	33%	0.756		
Femoral artery [n]	2	11%	6	15%	1.000		
Radial artery [n]	5	26%	4	10%	0.131		
Plaques	N	%	N	%	p-value		
Any plaques [n]	3	16%	15	36%	0.140		
Carotid Plaque [n]	3	16%	12	28%	0.356		
Femoral Plaque [n]	0	0%	8	23%	0.668		
Arterial stiffness	Mean	SD	Mean	SD	p-value		
CBSI	4.1	0.7	6.2	1.7	<0.001	-1.63[a]	-2.52;-0.76
CDC [%/10mmHg]	5.6	1.4	3.9	1.2	<0.001	1.27[a]	0.51;2.04

Supplemental Table 5. Comparison of vascular parameters between autologous HSCT and allogenic HSCT survivors, measurements of pulse wave velocities were unavailable for patients with autologous HSCT. AT – Adventitia thickness; BSA – Body-surface area; CBSI – Carotid β-stiffness index; CDC – Carotid distensibility coefficient; CI95% – 95% confidence interval; IMT – Intima-media thickness; LD – Lumen diameter; [a] – significant at 0.05-level; [b] – significant at p-0.005 level.

Supplemental Table 6. ANCOVA-models predicting measures of arterial stiffness among HSCT.

(A)

**Dependent variable**	**n**	**R^2**	**Model p-value**
Carotid β-Stiffness Index	60	0.275	<0.001
**Independent variables**	**β**	**CI95%**	**p-value**
Constant	1.73	-0.01;3.47	
Age [years]	0.09	0.04;0.15	0.004
LDL > 3.0mmol/l	0.82	0.02;1.63	0.044
TBI	1.13	0.24;2.01	0.014

(B)

**Dependent variable**	**n**	**R^2**	**Model p-value**
Carotid Distensbility Coefficient [%/10mmHg]	60	0.359	<0.001
**Independent variables**	**β**	**CI95%**	**p-value**
Constant	8.3	6.86;9.78	
Age [years]	-0.10	-0.14;0.06	<0.001
LDL > 3.0mmol/l	-0.91	-1.58;-0.24	0.009
TBI	-0.93	-1.67;-0.19	0.015

Supplemental Table 6. ANCOVA-models predicting (A) carotid artery β-stiffness index and (B) carotid artery distensibility coefficient. LDL – Low-density lipoprotein, TBI – Total body irradiation.

Supplemental figure 1. Scatter plots of carotid intima-media thickness among HSCT and controls

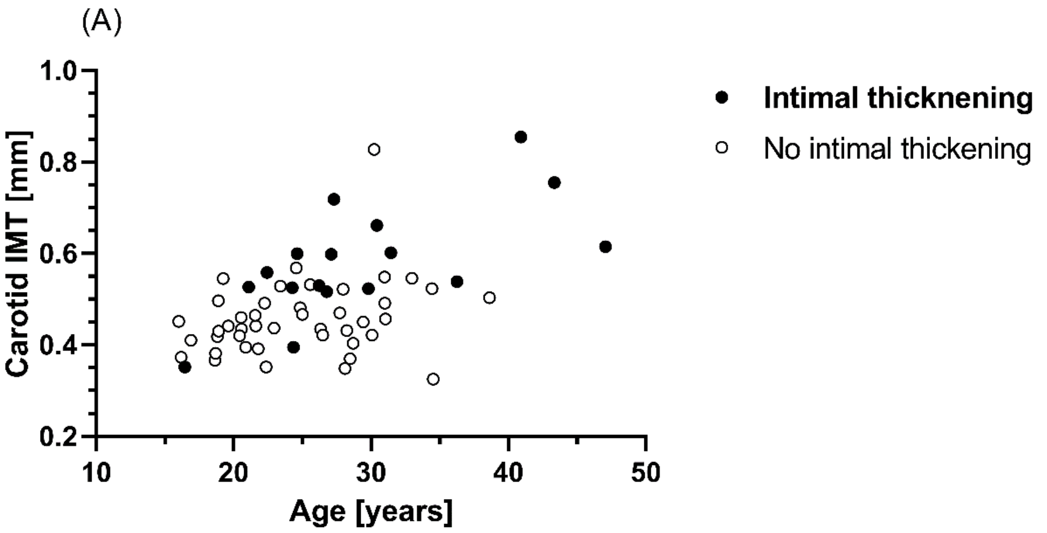

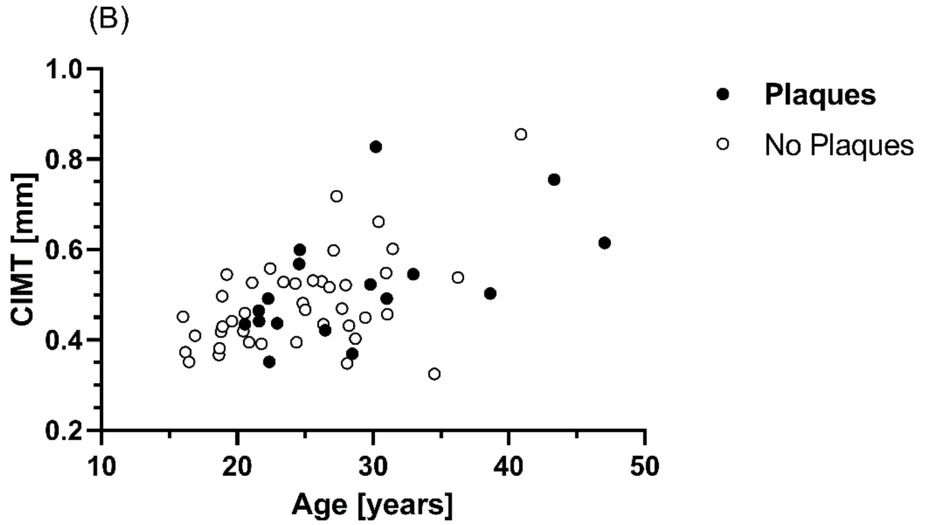

Supplemental figure 1. Scatter plots of (A) age and CIMT distribution in patients with and without VHRU-detected intimal thickening (B) and plaques in any arteries among HSCT patients. CIMT – Carotid artery intima-media thickness.
